# CIMP-positive glioma is associated with better prognosis: A systematic analysis

**DOI:** 10.1097/MD.0000000000030635

**Published:** 2022-09-30

**Authors:** Yingying Xu, Huashi Xiao, Wenqing Hu, He-Chun Shen, Wanjun Liu, Siyuan Tan, Chuanli Ren, Xiaomin Zhang, Xishuai Yang, Guo Yu, Ting Yang, Duonan Yu, Liang Zong

**Affiliations:** a Department of General Surgery, Yizhen People’s Hospital, Clinical Medical College, Yangzhou University, Yangzhou, Jiangsu Province, China; b Clinical Medical College, Dalian Medical University, Liaoning Province, China; c Department of Gastrointestinal Surgery, Changzhi People’s Hospital, The Affiliated Hospital of Changzhi Medical College, Changzhi, Shanxi Province, China; d Department of General Practice, Northern Jiangsu People’s Hospital, Clinical Medical College, Yangzhou University, Yangzhou, Jiangsu Province, China; e Department of Clinical Medical Testing Laboratory, Clinical Medical School of Yangzhou University, Northern Jiangsu People’s Hospital, Yangzhou, Jiangsu Province, China; f Central Laboratory, Changzhi People’s Hospital, The Affiliated Hospital of Changzhi Medical College, Changzhi, Shanxi Province, China; g Neurology Department, Changzhi People’s Hospital, The Affiliated Hospital of Changzhi Medical College, Changzhi, Shanxi Province, China; h Laboratory of Pharmacogenomics and Pharmacokinetic Research, Subei People’s Hospital, Yangzhou University, Yangzhou, Jiangsu Province, China; i Jiangsu Key Laboratory of Experimental & Translational Non-coding RNA Research, Yangzhou University School of Medicine, Yangzhou, Jiangsu Province, China; j Jiangsu Co-Innovation Center for Prevention and Control of Important Animal Infectious Disease and Zoonosis, Yangzhou, Jiangsu Province, China.

**Keywords:** CIMP (CpG island methylator phenotype), gene mutations, glioma, pathological features, prognosis, systematic review

## Abstract

CpG island methylator phenotype (CIMP) was closely related to the degree of pathological differentiation of tumors, and it’s an important determinant of glioma pathogenicity. However, the molecular and pathological features of CIMP-positive glioma have not been fully elucidated. In addition, CIMP have been reported to be a useful prognostic marker in several human cancers, yet its prognostic value in gliomas is still controversial. Therefore, we aimed to evaluate gene mutations and pathological features of CIMP-positive glioma and explore the prognostic value of CIMP in gliomas.

**Methods:**

We comprehensively searched PubMed, Embase, and MEDLINE for studies describing gene mutations, pathological features and overall survival of gliomas stratified by CIMP status. Odds ratios (OR), hazard ratios (HR), and their 95% confidence intervals (CI) were used to estimate the correlation between CIMP and the outcome parameters.

**Results:**

Twelve studies with 2386 gliomas (1051 CIMP-positive and 1335 CIMP-negative) were included. Our results showed that CIMP was more frequent in isocitrate dehydrogenase 1 (IDH1)-mutated gliomas (OR 229.07; 95% CI 138.72–378.26) and 1p19q loss of heterozygosis (LOH) gliomas (OR 5.65; 95% CI 2.66–12.01). Pathological analysis showed that CIMP was common in low-malignant oligodendroglioma (OR 5.51; 95% CI 3.95–7.70) with molecular features including *IDH1* mutations and 1p19q LOH, but rare in glioblastoma (OR 0.14; 95% CI 0.10–0.19). However, CIMP showed no obvious correlation with anaplastic oligoastrocytomas (OR 1.57; 95% CI 1.24–2.00) or oligoastrocytomas (OR 0.79; 95% CI 0.35–1.76). Concerning the prognosis, we found that CIMP-positive gliomas had longer overall survival (HR 0.57; 95% CI 0.97–0.16) than CIMP-negative gliomas.

**Conclusions:**

CIMP could be used as a potential independent prognostic indicator for glioma.

## 1. Introduction

Glioma is the most common malignant tumor of the central nervous system (CNS) and adversely affects human health with poor overall survival.^[1]^ In 2021, 24,530 new CNS cancers were reported in the United States, and 18,600 cases died of CNS cancer.^[2]^ Surgery combined with postoperative radiotherapy and chemotherapy remains the standard treatment for glioma. However, the average survival time for high-grade gliomas is still only approximately 15 months.^[3]^ With the development of molecular diagnosis and treatment technologies, prognostic markers play an increasingly important role in guiding clinical appropriate therapy and predicting tumor malignancy and prognosis.^[4]^ In 2016, the World Health Organization (WHO) CNS classification used molecular biomarkers to classify gliomas for the first time, such as isocitrate dehydrogenase 1 (IDH1), 1p19q loss of heterozygosity (LOH), and O6-methylguanine-DNA methyltransferase (MGMT) promoter methylation.^[5]^ In 2021, the WHO placed even more emphasis on molecular biomarkers.^[6]^ Such molecular alterations are crucial in understanding the classification, diagnosis, management, and prognosis of gliomas.^[7]^ However, the early diagnosis and prognosis of glioma are always a difficult point in the clinic. Thus, the identification of novel prognostic markers that are independent of the WHO molecular and histological tumor node metastasis staging systems for glioma are urgently needed.

CIMP, also called the CpG island methylator phenotype, has a high degree of methylation and is a distinct molecular characteristic of human cancer.^[8]^ Frequent abnormal CpG island deoxyribonucleic acid (DNA) methylation in the promoter region of certain genes is an important mechanism of epigenetic suppression that silences tumor-suppressive genes, and affects normal functions of cell proliferation and apoptosis, DNA repair and cell cycle regulation.^[9,10]^ Abnormal CIMP have been found in some aging normal cells and tumor cells such as gastric cancer and colorectal cancer.^[11]^ Through broad-spectrum gene methylation analysis, it was found that the mutation of the above-mentioned molecular genes were closely related to DNA methylation alteration in gliomas. Although DNA methylation-induced epigenetic changes induce carcinogenesis, the prognostic value of CIMP in most human cancers remains unclear.

Many studies have reported that CIMP was closely related to the degree of pathological differentiation of tumors, suggesting that it may be a powerful determinant of tumor pathogenicity in glioma.^[12,13]^ However, the molecular genetic characteristics, clinicopathological classification of CIMP-positive glioma have not been fully elucidated. Moreover, controversy surrounding the prognostic value of CIMP still exists in gliomas. Therefore, we aim to systematically analyze the relationship of between CIMP and gene mutations, pathological features, and prognosis in gliomas. To estimate the strength of this postulated relationship more accurately, the CIMP was determined to be either positive or negative, and we performed a systematic review and meta-analysis.

## 2. Methods

### 2.1. Search strategy

We performed a comprehensive literature search from the electronic databases, such as PubMed, Embase, and MEDLINE databases which focused on medicine, biomedical information and life science to identify relevant studies published up to February 2021. We utilized this search term combination: glioma and CIMP. Search results were combined in Endnote X8 to compile the reference manager database and duplicates were removed. Eligible studies were selected based on the inclusion and exclusion criteria. The reference lists of included studies were searched to identify other potential studies.

### 2.2. Study selection

The inclusion criteria were as follows: studies limited to human gliomas; studies evaluating the correlation between CIMP status and tumor node metastasis stage, gender, histology, molecular genes features or overall survival; sufficient published data to calculate the odds ratio (OR), hazard ratio (HR) and corresponding 95% confidence interval (CI). Furthermore, we excluded abstracts, editorials, letters, expert opinions, case reports, reviews, studies not written in English and impossible to extract the appropriate data.

### 2.3. Data extraction

Data extraction, based on the selection criteria, included the following information: last name of the first author, publication year, sample size, number of patients with positive CIMP, number of patients with negative CIMP, number of patients with IDH1, epidermal growth factor receptor (EGFR), MGMT, 1p19q LOH, gender, histology and overall survival in patients with and without CIMP. Outcomes were described as OR or HR with 95% CIs. We used the methods described by Tierney et al^[14]^ and Guyot et al^[15]^ to extract data from Kaplan–Meier curves.

### 2.4. Quality assessment

We assessed the quality of randomized and nonrandomized controlled trial studies using the Jadad Scale and the methodological index for non-randomized studies.^[16,17]^ These criteria are not fully demonstrated in molecular studies, therefore, we set strict criteria for the included studies, for example, we did not exclude the single-aim study specimen of glioma, discussed tumors at all stages, and no exclusion based on molecular markers.

### 2.5. Statistical analysis

All statistical tests were performed using Stata Version 13.0 (Stata Corporation, College Station, TX). ORs and their 95% CIs were used to assess the relationship between the CIMP status and pathological and molecular parameters. For the quantitative aggregation of survival results, HRs and their 95% CIs were both used as the effective values. The HRs were calculated directly from the reported data by the number of events or from the Kaplan–Meier survival curve using Engauge Digitizer software (freely downloaded from http://sourceforge.net). *χ*^2^-based *Q* test (*P *> .1 was considered a lack of heterogeneity), and *I*^2^ test (*I*^2^ ≤ 50% indicated low heterogeneity, and *I*^2^ > 50% indicated substantial heterogeneity) was calculated for an objective measure of heterogeneity between studies. For low-heterogeneity group, each study was analyzed using the fixed-effects model. Otherwise, the random effects model was used. The significance of the pooled OR or HR was determined by *Z* test (*P* < .05 was considered statistically significant). Funnel plots were presented to estimate potential publication bias, and an asymmetric plot suggested possible publication bias. Funnel plot asymmetry was evaluated by Egger linear regression test, which is a linear regression approach measuring funnel plot asymmetry on the OR natural logarithm scale. As suggested by Egger, significance of the intercept was determined by the *t* test (*P* < .05 considered representative of statistically significant publication bias).

## 3. Results

### 3.1. Study characteristics

**Figure 1.**
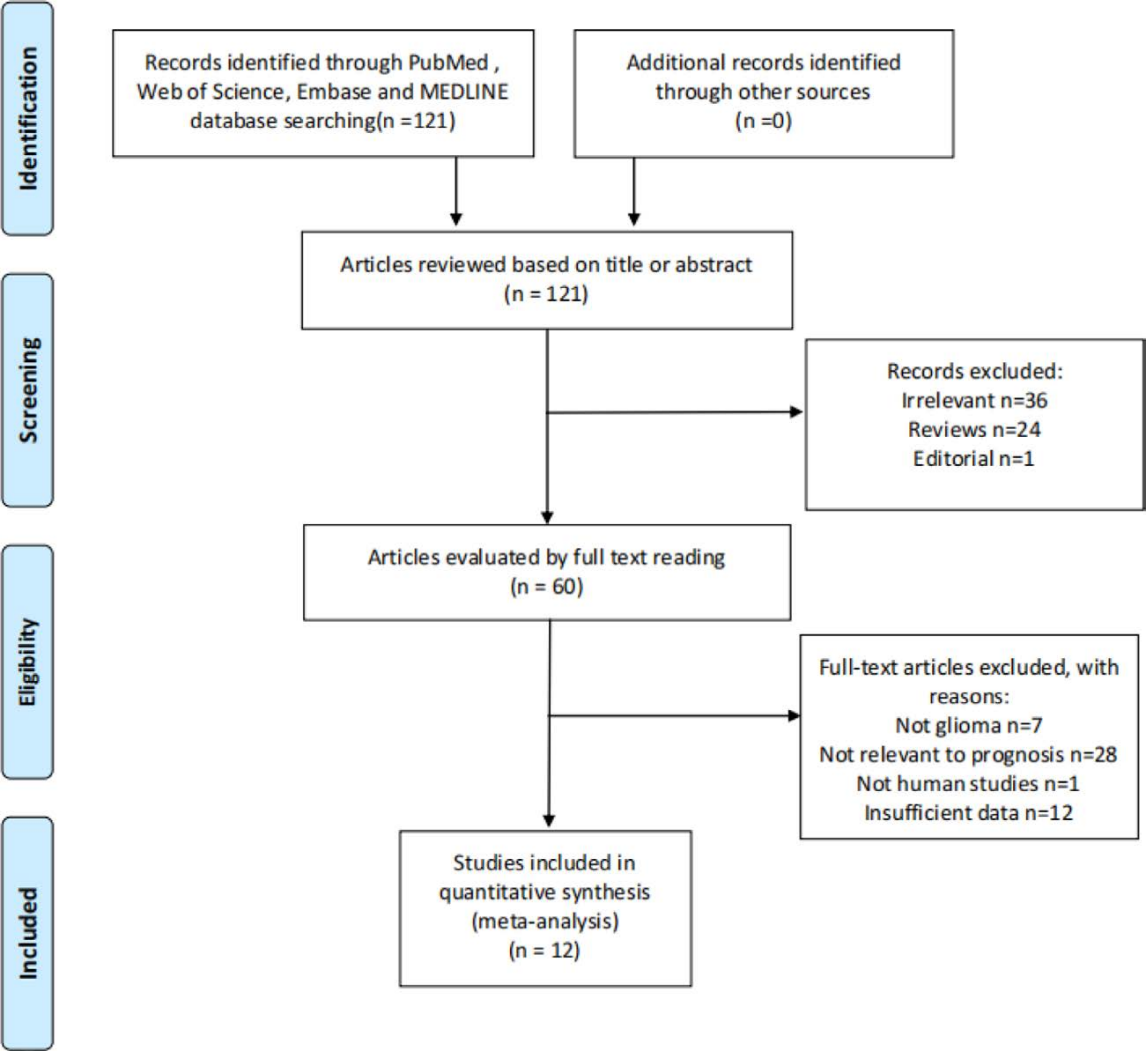
Flowchart of literature selection.

**Figure 2.**
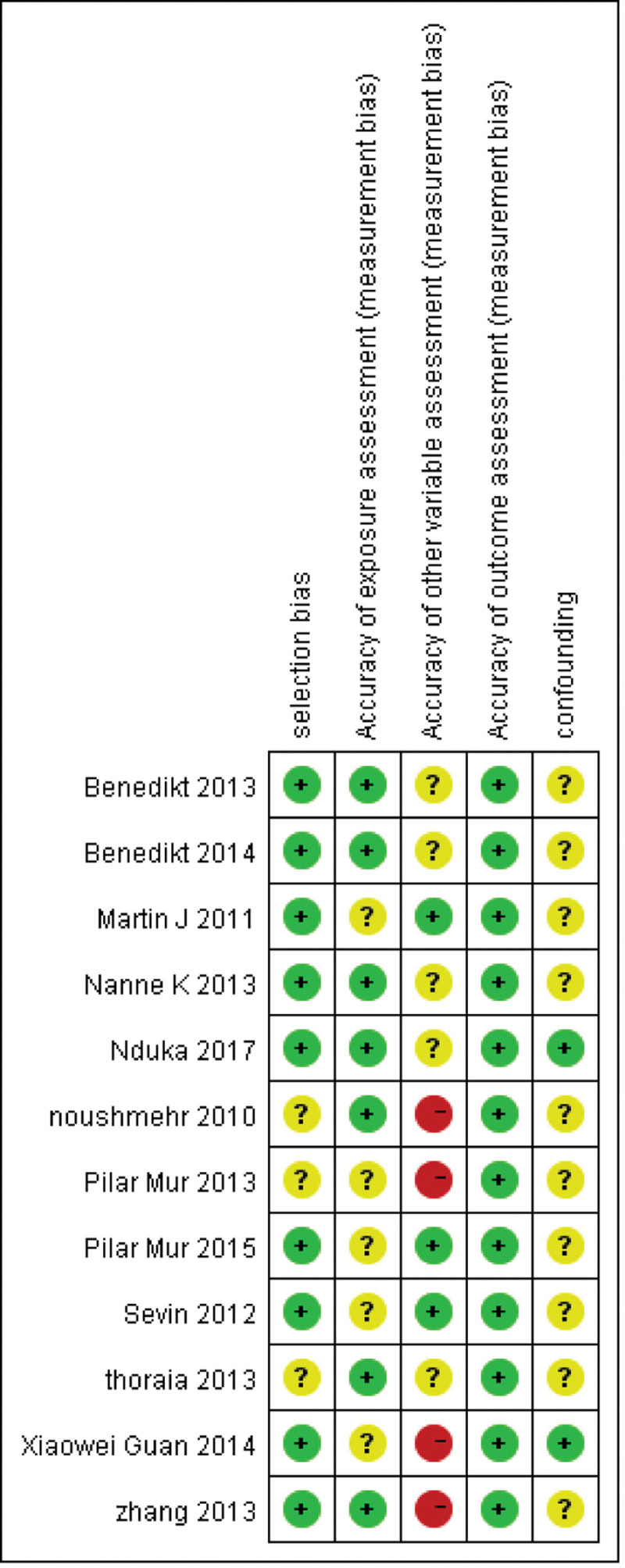
Risk of bias of each included study. Red cycle: study with high risk of bias; green cycle: study with low risk of bias; yellow cycle: study with insufficient information for assessing risk of bias.

The initial search from different databases yielded 121 potential articles. After title screening, abstract screening and full-text evaluation, 109 articles were excluded (Fig. 1). The 12 remaining studies included 2386 patients with 1051 CIMP-positive and 1335 CIMP-negative, where CIMP was classified in a dichotomized fashion (CIMP-positive vs negative) (Table 1).^[18–29]^ Among the 12 studies, all reported IDH1 mutations, 3 reported 1p19q LOH, 2 reported EGFR mutations, 2 reported MGMT promoter methylation, 3 reported gender, nine reported histology, and 5 reported overall survival. Sample size in the studies ranged from 33 to 1122. Four studies used samples from The Cancer Genome Atlas (TCGA),^[18,21,23,24]^ 2 from the Erasmus medical cancer brain tumor tissue bank,^[20,23]^ 2 from the Spanish National Tumor Bank Network,^[22,25]^ 2 from the Neurooncology Working Group trial in Germany,^[19,27]^ and 1 from the Chinese Glioma Genome Atlas.^[26]^ In addition, 1 study used data from a publicly available dataset^[29]^and another used mixed samples from 2 publicly available datasets and 1 newly generated dataset from MD Anderson.^[28]^ For each included study, Figure 2 summarized the risk of bias from selection, exposure assessment, outcome assessment, other variable assessment, and confounding factors. Based on strict exclusion and inclusion criteria, studies with high risk in selection bias were excluded.

**Table 1:** Summary of included study characteristics.

Study	Country	Patients population				CIMP assessment					Outcomes
		Sample size, N	Stage	Male gender	Median	Gene panel	Lab method	CIMP classification	Marker threshold	CIMP prevalence, N (%)	
Houtan Noushmehr et al^[18]^	USA	272	II-IV	NR	mean 56	DOCK5, ANKRD43,HFE, MAL, LGALS3, FAS-1, FAS-2, RHOF	MSP	CIMP±	CIMP+: ≥6/8	24 (8.8%)	OS
Martin J. van den Bent et al^[23]^	NR	68	NR	40 (58.5%)	<50y/o:N = 33; >=50y/o:N = 35	cl22, cl18, c19, c117, c123	MethyLight	CIMP±	CIMP+: HOPACH clustering red	31 (45.6%)	OS
Sevin Turcan et al^[24]^	USA	81	II-IV	48 (59.3%)	<40y/o:N = 23; 40-<50 y/o:N = 21;50-<60y/o:N = 18;>=60y/o:N = 19	PHC2, VIM, KIAA0494, SLC20A1, IFRD1, FABP5, DYNLT3, SPRY2, RUNX1, GRB10, SLC9A1, FNBP1, BCAT1, C2orf3, ITCH, MBNL3, ARNTL2	MSP	CIMP±	CIMP+: K -means consensus clustering red	49 (60.5%)	OS
Benedikt Wiestler et al^[19]^	German	126	NR	NR	>=65	DOCK5, ANKRD43, HFE, MAL, LGALS3, FAS-1, FAS-2, RHOF	MSP	CIMP±	CIMP+: DOCK5 hypomethylated and >=5/remaining 7	7 (5.6%)	OS, EFS
Nanne K. Kloosterhof et al^[20]^	NR	138	II-IV	87 (63.0%)	mean 44	IGS-16, IGS-18, IGS-23, IGS-17, IGS-9	MSP	CIMP±	CIMP+: HOPACH clustering red	85 (61.6%)	NR
Thoraia Shinawi et al^[21]^	NR	35	IV	21 (60%)	NR	ANKRD43, DOCK5, LGALS3, FAS, HFE, MAL, RHOF	MSP	CIMP±	CIMP+: ≥3/7	5 (14.3%)	OS
Wei Zhang et al^[26]^	China	33	IV	22 (59.42%)	NR	ANKRD43, DOCK5, HFE, MAL, LGALS3, FAS-1, FAS-2, RHOF	MethyLight	CIMP±	CIMP+: consensus cluster 1 (n = 12 tumors) red	6 (18.2%)	NR
Benedikt Wiestler et al^[27]^	German	115	NR	NR	mean 42	ANKRD43, DOCK5, HFE, MAL, LGALS3, FAS-1, FAS-2, RHOF	MSP	CIMP±	CIMP+:DOCK5 hypomethylated and >=5/remaining 7	91 (79.1%)	OS, PFS
Xiaowei Guan et al^[28]^	USA	716	II-IV	NR	NR	The Verhaak 840-gene	MSP	CIMP±	CIMP+:>=1/5	208(40.9%)	OS
Nduka M. Amankulor et al^[29]^	USA	500	II-III	NR	NR	DOCK5, ANKRD43, HFE, MAL, LGALS3, FAS-1, FAS-2, RHOF	MSP	CIMP±	CIMP+: 1503 CIMP classifier probes red	419 (83.8%)	NR
Pilar Mur et al^[22]^	Spanish	55	II-III	31 (56.4%)	mean 48	TRIP4, DRG2, ASL, C1orf64, FLJ11286, CRELD1	MethyLight	CD-CIMP+/CIMP+/CIMP-	CD-CIMP+: 24.7% total probes; CIMP+: 21.4% total probes	38 (69.1%)	OS
Pilar Mur et al^[25]^	USA	247	II-IV	NR	<65y/o:N = 134;>=65y/o:N = 53	CpG165, CpG25, STP27	MSP	CIMP±	CIMP+:CpG165 methylation	88 (35.6%)	OS

CD-CIMP+ = codeleted-CIMP+, CIMP = CpG island methylator phenotype, EFS = events-free survival, Hopach = hierarchical ordered partitioning and collapsing hybrid, MSP = methylation-specific polymerase chain reaction, NR = not reported, OS = overall survival, PFS = progression-free survival, y/o = years old.

### 3.2. Gene mutations

**Figure 3.**
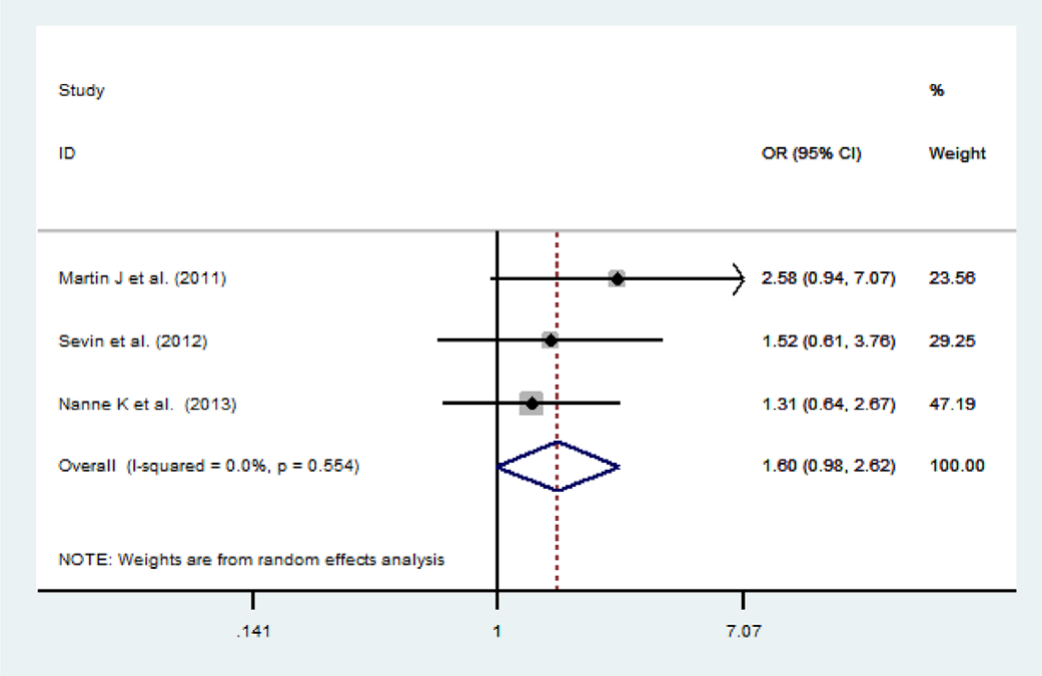
Meta-analysis of studies to investigate the molecular features of glioma patients associated with CIMP. CI = confidence interval, CIMP = CpG island methylator phenotype, EGFR = epidermal growth factor receptor, IDH1 = isocitrate dehydrogenase 1, LOH = 1oss of heterozygosis, MGMT = O^6^-methylguanine-DNA methyltransferase, OR = odds ratio.

The present research suggests that *IDH1* mutation, 1p19q LOH, *EGFR* mutation and *MGMT* promoter methylation are newly added major molecular markers for genetic molecular typing of glioma. For the purpose of pooled analysis, CIMP + (CIMP-positive) glioma was compared with CIMP- (CIMP-negative) glioma. The pooled OR for *IDH1* mutation in the CIMP + versus CIMP- glioma revealed a significantly higher risk of *IDH1* mutation in the CIMP + glioma (OR 229.07; 95% CI 138.72–378.26; *P* < .00001, *P*_*heterogeneity*_ 0.000). Similarly, a higher risk of 1p19q LOH was observed in CIMP + glioma (OR 5.65; 95% CI 2.66–12.01; *P* = .01, *P*_*heterogeneity*_ 0.040), whereas *EGFR* mutation and *MGMT* promoter methylation did not show any differences between the 2 types of gliomas [(OR 0.14; 95% CI 0.05–0.43; *P* = .35; *P*_*heterogeneity*_ 0.002) and (OR 3.01; 95% CI 0.79–11.48; *P* = .10; *P*_*heterogeneity*_ 0.825)] (Fig. 3).

### 3.3. Pathological features

**Figure 4.**
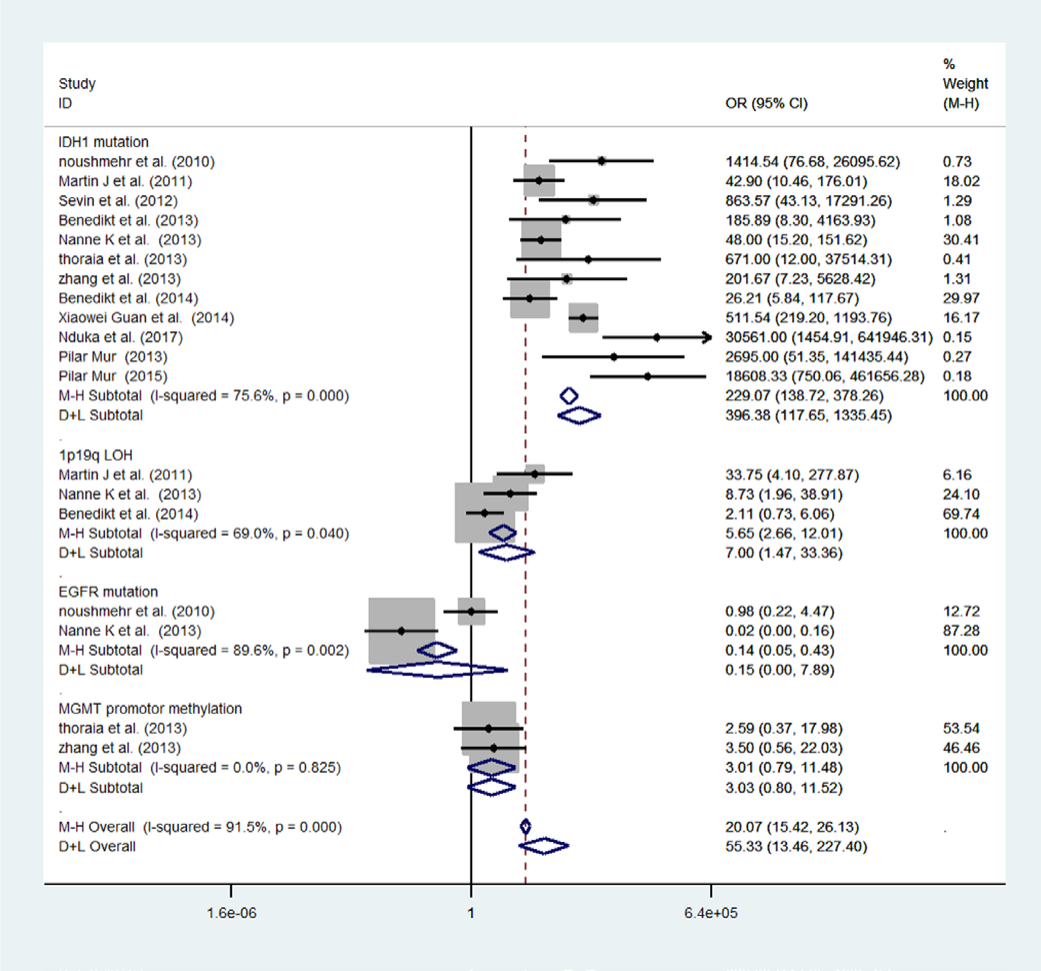
Meta-analysis of studies to investigate the gender of glioma patients associated with CIMP. CI = confidence interval, CIMP = CpG island methylator phenotype, OR = odds ratio.

**Figure 5.**
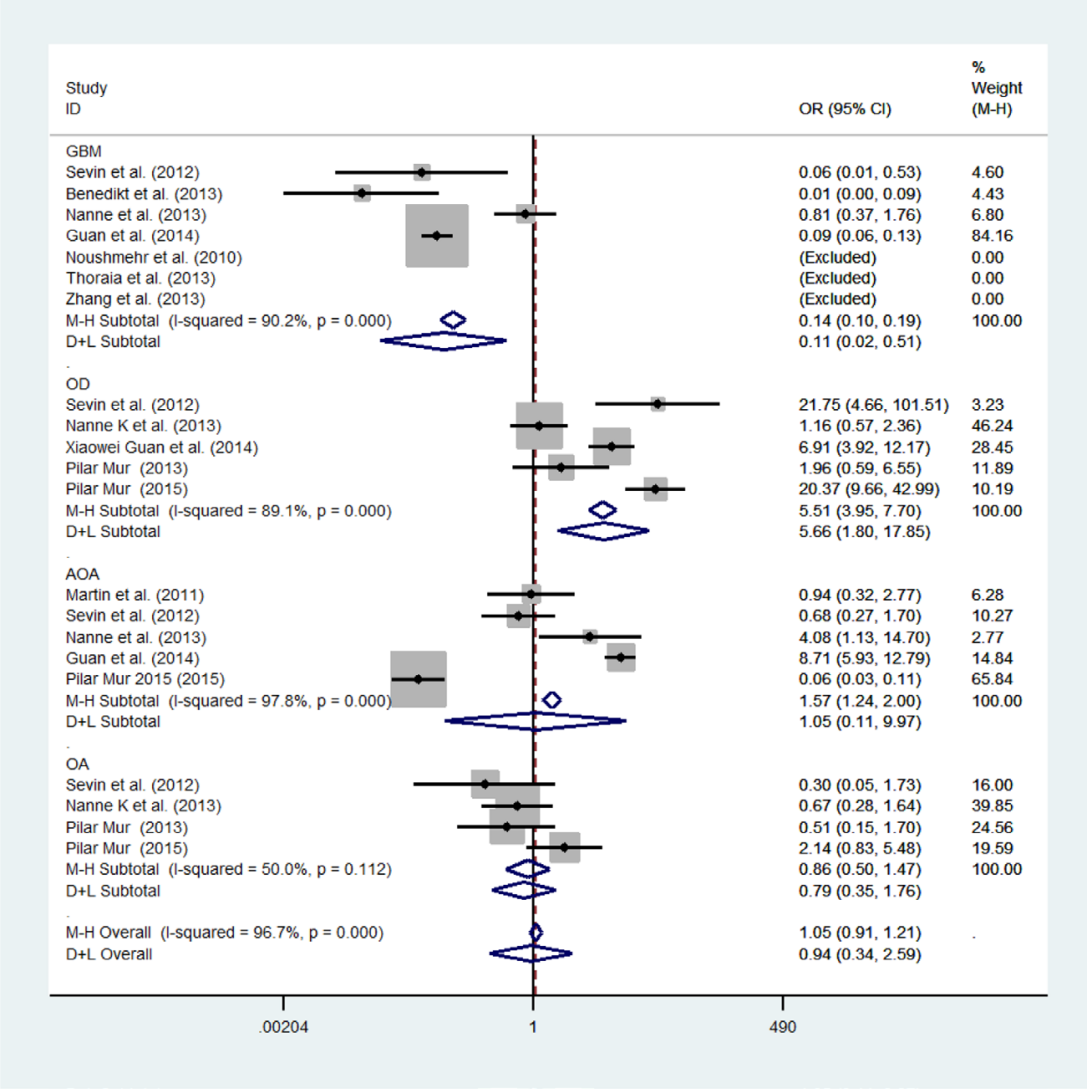
Meta-analysis of studies to investigate the histopathology features of glioma patients related with CIMP. AOA = anaplastic oligoastrocytomas, CI = confidence interval, CIMP = CpG island methylator phenotype, GBM = glioblastoma, OA = oligoastrocytoma, OD = oligodendroglioma, OR = odds ratio.

Extractable data related to pathological factors were gender and histopathology. The overall OR for the proportions of males in CIMP + versus CIMP- gliomas was 1.60 (95% CI 0.98–2.62; *P* = .06; *P*_*heterogeneity*_ 0.554; Fig. 4). The International Classification of Diseases for Oncology (ICD-O) is the most important standard classification in clinical medicine, and is used by all medical professionals. The ICD-O topography codes largely correspond to those in the tenth edition of the International Statistical Classification of Diseases, Injuries, and Causes of Death (ICD-10).^[5,30]^ WHO classified gliomas into low-grade gliomas (LGG) with low malignancy and high-grade gliomas. Compared to high-grade gliomas, such as anaplastic oligoastrocytomas (AOA, WHO Grade III, ICD-O 9382/3) or glioblastoma (GBM, WHO Grade IV, ICD-O 9440/3), LGG including oligoastrocytomas (OA, WHO Grade II, ICD-O 9382/3) and oligodendroglioma (OD, WHO Grade II, ICD-O 9450/3) had better overall survival prognosis.^[30]^ Our analysis showed that GBM and OD in the CIMP + and CIMP- groups achieved statistical significance [(OR 0.14; 95% CI 0.10–0.19; *P *= .005, *P*_*heterogeneity*_ 0.000) and (OR 5.51; 95% CI 3.95–7.70; *P* = .003, *P*_*heterogeneity*_ 0.000)], whereas no differences were shown for AOA and OA [(OR 1.57; 95% CI 1.24-2.00; *P* = .97; *P*_*heterogeneity*_ 0.000) and (OR 0.79; 95% CI 0.35–1.76; *P* = .54; *P*_*heterogeneity*_ 0.112; Fig. 5)].

### 3.4. Overall survival and publication bias

**Figure 6.**
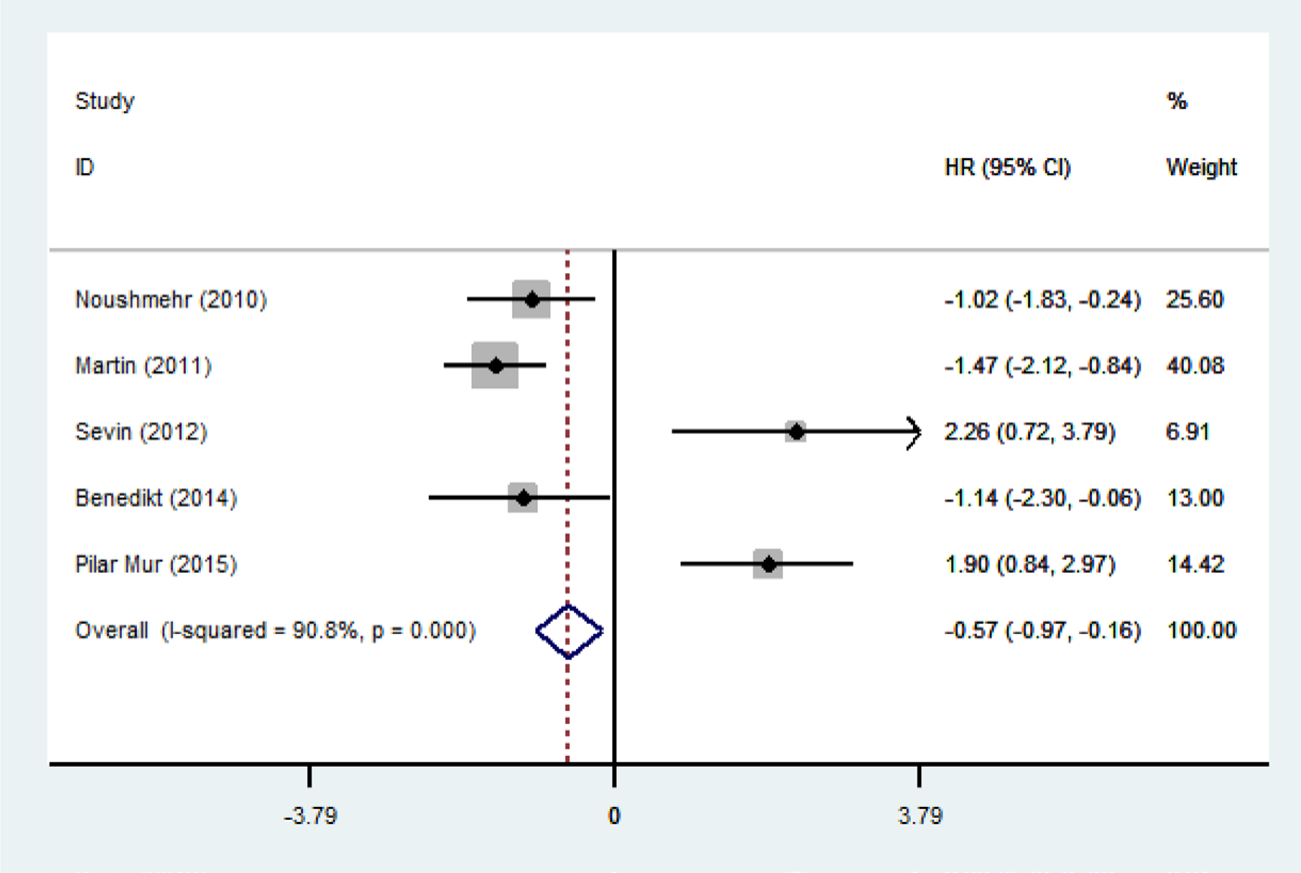
Meta-analysis of overall survival (OS) in studies of CIMP-positive versus CIMP-negative gliomas. Random effect meta-analysis showing longer OS in CIMP-positive gliomas. CI = confidence interval, CIMP = CpG island methylator phenotype, HR = hazard ratio.

The prognostic role of CIMP status has been evaluated in various tumors, such as colorectal cancer, hepatocellular carcinoma and gastric cancer.^[31–33]^ In order to investigate whether CIMP status has prognostic value in glioma, we pooled 5 studies that has completed the correlation analysis between the overall survival of individuals and CIMP + or CIMP- gliomas. We found that CIMP + glioma was significantly associated with longer overall survival (HR 0.57; 95% CI 0.97-0.16; *P* = .003; *P*_*heterogeneity*_ 0.000; Fig. 6).

Begg funnel plot was used to assess publication bias. Heterogeneity comparison of 12 combined studies showed that heterogeneity existed in certain analyses such as *IDH1* mutation, 1p19q LOH, *EGFR* mutation, AOA, OD, GBM, and overall survival. However, no single study influenced the pooled OR qualitatively as indicated by the sensitivity analyses (data not shown).

## 4. Discussion

Epigenetic alterations have been reported to be involved in the process of tumor carcinogenesis through various mechanisms such as histone modifications, DNA methylation, small and long noncoding ribonucleic acid, and chromatin architecture remodeling.^[34]^ With the unclear significant impact of aberrant DNA sequence changes in human cancers and the irreversible and hereditary characteristics of epigenetic alterations,^[35]^ the presence of epigenetic alterations in noncancerous tissues suggests that epigenetic alterations are involved in the field of cancerization. CIMP is 1 of the most reported epigenetic alterations and is recognized as a major event in the origin of many cancers.^[8,34]^

Prognostic value of CIMP in a variety of tumors has been reported.^[36,37]^ For example, global genome hypermethylation, resulting in the switch off of tumor suppressor genes, indicated as CIMP, which is closely associated with a worse outcome in colorectal cancer.^[31,38]^ In addition, CIMP, an indicator of poor prognosis, is related to a higher mutation burden of *bromodomain-containing protein*, *DNA damage-induced apoptosis suppressor*, and *nicotinamide adenine dinucleotide phosphate oxidase 1* in hepatocellular carcinoma patients.^[32]^ CIMP is also a potential biomarker for the treatment of patients with gastric cancer.^[33]^ However, the prognostic role of the CIMP status in gliomas is uncertain. In this study, we expanded upon previous tumor-associated research on the prognostic value of CIMP to examine CpG islands associated with glioma. Several researches have suggested that the potential cancer-specific mutated driver genes include *IDH1/2* and *H3 histone* (*H3F3A*).^[39]^

It has been well documented that CIMP of genes is associated with survival in glioma patients. The ADP-ribosylation factor family plays an oncogenic role in the development of gliomas. One study showed that AL9R hypermethylation can predict favorable OS and progression-free survival in patients with LGG and it could be as a prognostic biomarker for LGG.^[40]^ In addition, the TP73 gene encodes protein 73. Chen et al confirmed that 8 methylation sites of TP73 gene CpG island were significantly positively associated with better OS and progression-free survival of patients diagnosed with grade II/III glioma.^[41]^ EMILIN2 is an extracellular matrix protein, and LGG data from TCGA discovered that the EMILIN2 expression, negatively correlated to the EMILIN2 methylation, could predict a poor prognosis.^[42]^ However, another research found that the inhibition of methylated miR-338-5p-5p in the promoter region was related to AOA invision.^[43]^ It was because of these inconsistent prognostic results that we further used a meta-analysis to research the prognostic value of CIMP in gliomas.

We identified 12 published studies, including 2386 glioma patients to assess the correlation between CIMP and gene mutations or pathological features in gliomas. More *IDH1* mutations, 1p19q LOH and OD, and less GBM were found in CIMP-positive glioma than in CIMP-negative glioma. Moreover, we also identified that CIMP did not show a correlation with *MGMT* promoter methylation, *EGFR* mutation, AOA, OA or gender, but CIMP-positive was significantly associated with longer overall survival. Taken together, these results suggest that CIMP may be used as an independent prognostic marker in glioma patients.

Heterogeneity in the relationship between the CIMP status and certain pathological features was significant in this study. One of the main confounding factors of significant heterogeneity may be the lack of a standardized definition of CIMP, with the number, type and identity of genes employed in the selection panel different in each study. Until 2010, Noushmeh^[18]^ reported that in 272 gliomas in the context of TCGA, 3 DNA methylation clusters were identified by GoledenGate and Infinium data. Cluster 1 formed a highly characteristic DNA methylation profile, showing GBM-specific methylation changes at a subset of loci, which was designated as the glioma-CIMP (G-CIMP). Further, Noushmeh and colleagues validated that 8 genes were formed at the G-CIMP loci. A sample was considered G-CIMP-positive when 7 loci (*ANKRD43*, *HFE*, *MAL*, *LGALS3*, *FAS-1*, *FAS-2*, and *RHO-F*) were hypermethylated and 1 locus, *DOCK5*, was hypomethylated.^[18]^ Moreover, they also demonstrated that the G-CIMP status was more common in the grade II and III glioma with improved survival.^[18]^ Until recently, classifications based on CIMP-positive versus CIMP-negative, as well as classifications based on *IDH1*-mutant (G-CIMP-high, G-CIMP-low subgroups), were widely used in a variety of studies for glioma.^[35,44]^ Further studies are needed to verify a consistent CIMP definition.

So far, the value of CIMP as a predictive biomarker to guide the prescription of neoadjuvant or adjuvant chemotherapy in glioma is uncertain. However, considering the influence of CIMP in therapeutic and clinical trial strategy may be necessary. It is clear that there is heterogeneity, even within other molecule biomarker combinations, which is likely to lead to potential prognostic value for individualized therapy.^[45]^ Malta^[39]^ reported that glioma were divided into 2 clinically relevant subsets (CIMP-high, CIMP-low). Their research showed that *IDH1* mutation and CIMP were independent predictors of outcome, suggesting that CIMP and *IDH1* mutation are potential prognostic biomarkers in glioma. Furthermore, G-CIMP tumor-related genes exhibited a demethylated pattern, and reversing the methylated pattern of G-CIMP tumor-related genes may be a potential solution for glioma.^[46,47]^ Further work should be conducted to verify and confirm the clinical value of CIMP in patients with glioma.

The main limitation of our research was the spectrum of gene panel markers used for CIMP. In fact, this is a common finding in CIMP studies, and other systematic reviews and meta-analyses on gastric cancer^[33,48]^ and colorectal cancer^[49]^ also have accepted this relative limitation in pooled analyses. This study has great advantages because it was a systematic review and meta-analysis of the currently available literature on the prognostic value of CIMP in gliomas.

## 5. Conclusions

In conclusion, this meta-analysis highlights that there are specific molecular (such as *IDH1* mutations and 1p19q LOH) and pathological features and a better prognosis in CIMP-positive gliomas, suggesting that CIMP could be used as an independent prognostic marker for glioma.

## Author contributions

**Conceptualization:** Liang Zong.

**Data curation:** Yingying Xu, Huashi Xiao, Wenqing Hu.

**Formal analysis:** Huashi Xiao, Wenqing Hu.

**Funding acquisition:** Duonan Yu.

**Investigation:** Siyuan Tan, Chuanli Ren.

**Methodology:** Yingying Xu, Huashi Xiao.

**Supervision:** Duonan Yu.

**Validation:** He-Chun Shen, Wanjun Liu.

**Visualization:** Siyuan Tan.

**Writing – original draft:** Yingying Xu, Duonan Yu.

**Writing – review & editing:** He-chun Shen, Wanjun Liu, Siyuan Tan, Chuanli Ren, Xiaomin Zhang, Xishuai Yang, Guo Yu, Ting Yang.

## References

[1] Markouli M Strepkos D Papavassiliou KA. Bivalent genes targeting of glioma heterogeneity and plasticity. Int J Mol Sci . 2021;22:540. 10.3390/ijms22020540 PMC7826605 33430434

[2] Siegel RL Miller KD Fuchs HE. Cancer statistics, 2021. CA Cancer J Clin. 2021;71:7–33. 33433946 10.3322/caac.21654

[3] Hombach-Klonisch S Mehrpour M Shojaei S. Glioblastoma and chemoresistance to alkylating agents: involvement of apoptosis, autophagy, and unfolded protein response. Pharmacol Ther. 2018;184:13–41. 29080702 10.1016/j.pharmthera.2017.10.017

[4] Sledzinska P Bebyn MG Furtak J. Prognostic and predictive biomarkers in gliomas. Int J Mol Sci. 2021;22:10373. 34638714 10.3390/ijms221910373 PMC8508830

[5] Louis DN Perry A Reifenberger G. The 2016 World Health Organization classification of tumors of the central nervous system: a summary. Acta Neuropathol. 2016;131:803–20. 27157931 10.1007/s00401-016-1545-1

[6] Louis DN Perry A Wesseling P. The 2021 WHO classification of tumors of the central nervous system: a summary. Neuro Oncol. 2021;23:1231–51. 34185076 10.1093/neuonc/noab106 PMC8328013

[7] Liu J Gao L Ji B. BCL7A as a novel prognostic biomarker for glioma patients. J Transl Med. 2021;19:335. 34362400 10.1186/s12967-021-03003-0 PMC8348860

[8] Advani SM Swartz MD Loree J. Epidemiology and molecular-pathologic characteristics of CpG Island methylator phenotype (CIMP) in colorectal cancer. Clin Colorectal Cancer. 2021;20:137–147.e1. 33229221 10.1016/j.clcc.2020.09.007

[9] Skvortsova K Masle-Farquhar E Luu PL. DNA Hypermethylation encroachment at CpG Island borders in cancer is predisposed by H3K4 monomethylation patterns. Cancer Cell. 2019;35:297–314.e8. 30753827 10.1016/j.ccell.2019.01.004

[10] Ma L Huang Y Zhang H. Sensitive detection and conjoint analysis of promoter methylation by conjugated polymers for differential diagnosis and prognosis of glioma. ACS Appl Mater Interfaces. 2021;13:9291–9. 32436715 10.1021/acsami.0c03218

[11] Toyota M Ahuja N Ohe-Toyota M. CpG island methylator phenotype in colorectal cancer. Proc Natl Acad Sci USA. 1999;96:8681–6. 10411935 10.1073/pnas.96.15.8681 PMC17576

[12] Weng JY Salazar N. DNA methylation analysis identifies patterns in progressive glioma grades to predict patient survival. Int J Mol Sci. 2021;22:1020. 33498463 10.3390/ijms22031020 PMC7864199

[13] Court F Le Boiteux E Fogli A. Transcriptional alterations in glioma result primarily from DNA methylation-independent mechanisms. Genome Res. 2019;29:1605–21. 31533980 10.1101/gr.249219.119 PMC6771409

[14] Tierney JF Stewart LA Ghersi D. Practical methods for incorporating summary time-to-event data into meta-analysis. Trials. 2007;8:16. 17555582 10.1186/1745-6215-8-16 PMC1920534

[15] Guyot P Ades AE Ouwens MJ. Enhanced secondary analysis of survival data: reconstructing the data from published Kaplan-Meier survival curves. BMC Med Res Methodol. 2012;12:9. 22297116 10.1186/1471-2288-12-9 PMC3313891

[16] Jadad AR Moore RA Carroll D. Assessing the quality of reports of randomized clinical trials: is blinding necessary? Control Clin Trials. 1996;17:1–12. 8721797 10.1016/0197-2456(95)00134-4

[17] Slim K Nini E Forestier D. Methodological index for non-randomized studies (minors): development and validation of a new instrument. ANZ J Surg. 2003;73:712–6. 12956787 10.1046/j.1445-2197.2003.02748.x

[18] Noushmehr H Weisenberger DJ Diefes K. Identification of a CpG island methylator phenotype that defines a distinct subgroup of glioma. Cancer Cell. 2010;17:510–22. 20399149 10.1016/j.ccr.2010.03.017 PMC2872684

[19] Wiestler B Claus R Hartlieb SA. Malignant astrocytomas of elderly patients lack favorable molecular markers: an analysis of the NOA-08 study collective. Neuro Oncol. 2013;15:1017–26. 23595628 10.1093/neuonc/not043 PMC3714152

[20] Kloosterhof NK de Rooi JJ Kros M. Molecular subtypes of glioma identified by genome-wide methylation profiling. Genes Chromosomes Cancer. 2013;52:665–74. 23629961 10.1002/gcc.22062

[21] Shinawi T Hill VK Krex D. DNA methylation profiles of long- and short-term glioblastoma survivors. Epigenetics. 2013;8:149–56. 23291739 10.4161/epi.23398 PMC3592900

[22] Mur P Mollejo M Ruano Y. Codeletion of 1p and 19q determines distinct gene methylation and expression profiles in IDH-mutated oligodendroglial tumors. Acta Neuropathol. 2013;126:277–89. 23689617 10.1007/s00401-013-1130-9

[23] van den Bent MJ Gravendeel LA Gorlia T. A hypermethylated phenotype is a better predictor of survival than MGMT methylation in anaplastic oligodendroglial brain tumors: a report from EORTC study 26951. Clin Cancer Res. 2011;17:7148–55. 21914791 10.1158/1078-0432.CCR-11-1274

[24] Turcan S Rohle D Goenka A. IDH1 mutation is sufficient to establish the glioma hypermethylator phenotype. Nature. 2012;483:479–83. 22343889 10.1038/nature10866 PMC3351699

[25] Mur P Rodriguez de Lope A Diaz-Crespo FJ. Impact on prognosis of the regional distribution of MGMT methylation with respect to the CpG island methylator phenotype and age in glioma patients. J Neurooncol. 2015;122:441–50. 25682093 10.1007/s11060-015-1738-9

[26] Zhang W Yan W You G. Genome-wide DNA methylation profiling identifies ALDH1A3 promoter methylation as a prognostic predictor in G-CIMP- primary glioblastoma. Cancer Lett. 2013;328:120–5. 22960273 10.1016/j.canlet.2012.08.033

[27] Wiestler B Capper D Hovestadt V. Assessing CpG island methylator phenotype, 1p/19q codeletion, and MGMT promoter methylation from epigenome-wide data in the biomarker cohort of the NOA-04 trial. Neuro Oncol. 2014;16:1630–8. 25028501 10.1093/neuonc/nou138 PMC4232086

[28] Guan X Vengoechea J Zheng S. Molecular subtypes of glioblastoma are relevant to lower grade glioma. PLoS One. 2014;9:e91216. 24614622 10.1371/journal.pone.0091216 PMC3948818

[29] Amankulor NM Kim Y Arora S. Mutant IDH1 regulates the tumor-associated immune system in gliomas. Genes Dev. 2017;31:774–86. 28465358 10.1101/gad.294991.116 PMC5435890

[30] Louis DN Ohgaki H Wiestler OD. The 2007 WHO classification of tumours of the central nervous system. Acta Neuropathol. 2007;114:97–109. 17618441 10.1007/s00401-007-0243-4 PMC1929165

[31] Zong L Abe M Ji J. Tracking the correlation between CpG Island methylator phenotype and other molecular features and clinicopathological features in human colorectal cancers: a systematic review and meta-analysis. Clin Transl Gastroenterol. 2016;7:e151. 26963001 10.1038/ctg.2016.14 PMC4822093

[32] Li G Xu W Zhang L. Development and validation of a CIMP-associated prognostic model for hepatocellular carcinoma. EBioMedicine. 2019;47:128–41. 31492561 10.1016/j.ebiom.2019.08.064 PMC6796541

[33] Powell A Soul S Christian A. Meta-analysis of the prognostic value of CpG island methylator phenotype in gastric cancer. Br J Surg. 2018;105:e61–8. 29341152 10.1002/bjs.10742

[34] Grady WM. Epigenetic alterations in the gastrointestinal tract: current and emerging use for biomarkers of cancer. Adv Cancer Res. 2021;151:425–68. 34148620 10.1016/bs.acr.2021.02.006

[35] Esteve-Codina A Alameda F Carrato C. RNA sequencing and immunohistochemistry reveal ZFN7 as a stronger marker of survival than molecular subtypes in G-CIMP-negative glioblastoma. Clin Cancer Res. 2021;27:645–55. 33106291 10.1158/1078-0432.CCR-20-2141

[36] Ning G Li Y Chen W. CpG Island methylator phenotype modulates the immune response of the tumor microenvironment and influences the prognosis of pancreatic cancer patients. J Oncol. 2021;2021:2715694. 34876903 10.1155/2021/2715694 PMC8645373

[37] Liang J Liu T Liao J. Development and validation of a CpG island methylator phenotype-related prognostic signature for cholangiocarcinoma. J Cell Physiol. 2021;236:3143–56. 32996133 10.1002/jcp.30082

[38] Nazemalhosseini Mojarad E Kuppen PJ Aghdaei HA. The CpG island methylator phenotype (CIMP) in colorectal cancer. Gastroenterol Hepatol Bed Bench. 2013;6:120–8. 24834258 PMC4017514

[39] Malta TM de Souza CF Sabedot TS. Glioma CpG island methylator phenotype (G-CIMP): biological and clinical implications. Neuro Oncol. 2018;20:608–20. 29036500 10.1093/neuonc/nox183 PMC5892155

[40] Tan Y Zhang S Xiao Q. Prognostic significance of ARL9 and its methylation in low-grade glioma. Genomics. 2020;112:4808–16. 32882327 10.1016/j.ygeno.2020.08.035 PMC7462573

[41] Chen Y Wang Y He Q. Integrative analysis of TP73 profile prognostic significance in WHO grade II/III glioma. Cancer Med. 2021;10:4644–57. 34121368 10.1002/cam4.4016 PMC8267133

[42] Wang LC Cui WY Zhang Z. Expression, methylation and prognostic feature of EMILIN2 in Low-Grade-Glioma. Brain Res Bull. 2021;175:26–36. 34280481 10.1016/j.brainresbull.2021.07.013

[43] Wang J Huo C Yin J. Hypermethylation of the promoter of miR-338-5p mediates aberrant expression of ETS-1 and is correlated with disease severity of astrocytoma patients. Front Oncol. 2021;11:773644. 34858853 10.3389/fonc.2021.773644 PMC8632532

[44] Ruiz-Rodado V Malta TM Seki T. Metabolic reprogramming associated with aggressiveness occurs in the G-CIMP-high molecular subtypes of IDH1mut lower grade gliomas. Neuro Oncol. 2020;22:480–92. 31665443 10.1093/neuonc/noz207 PMC7158660

[45] Ziogas DC Dimopoulos MA Kastritis E. Prognostic factors for multiple myeloma in the era of novel therapies. Expert Rev Hematol. 2018;11:863–79. 30334460 10.1080/17474086.2018.1537776

[46] Yin AA He YL Etcheverry A. Novel predictive epigenetic signature for temozolomide in non-G-CIMP glioblastomas. Clin Epigenetics. 2019;11:76. 31088577 10.1186/s13148-019-0670-9 PMC6515684

[47] Moure CJ Diplas BH Chen LH. CRISPR editing of mutant IDH1 R132H induces a CpG methylation-low state in patient-derived glioma models of G-CIMP. Mol Cancer Res. 2019;17:2042–50. 31292202 10.1158/1541-7786.MCR-19-0309 PMC6774824

[48] Reynolds IS Furney SJ Kay EW. Meta-analysis of the molecular associations of mucinous colorectal cancer. Br J Surg. 2019;106:682–91. 30945755 10.1002/bjs.11142

[49] Jia M Gao X Zhang Y. Different definitions of CpG island methylator phenotype and outcomes of colorectal cancer: a systematic review. Clin Epigenetics. 2016;8:25. 26941852 10.1186/s13148-016-0191-8 PMC4776403

